# Evaluation of custom-made 3D printed polylactic acid/polyethylene glycol scaffolds in soft tissue augmentation: an experimental study in a canine model

**DOI:** 10.1186/s12903-025-07051-6

**Published:** 2025-10-27

**Authors:** Eman El-Mahalawy, Fatma Ramzy Kamel, Maha R. Taalab, Moustafa Aboushleib, Azza Saleh Koura, Wessam M. Amin

**Affiliations:** 1 Oral Medicine, Periodontology, Oral Diagnosis and Radiology Department, Faculty of Dentistry, Alexandria University, Champolion St. Azarita, Alexandria, 21521 Egypt; 2 Department of Dental Materials, Faculty of Dentistry, Alexandria University, Champolion St. Azarita, Alexandria, 21521 Egypt; 3 Department of Oral Biology, Faculty of Dentistry, Alexandria University, Champolion St. Azarita, Alexandria, 21521 Egypt; 4 Department of Materials Science, Institute of Graduate Studies and research, Alexandria University, 163 Horrya Road Elshatby, Alexandria, 21526 Egypt

**Keywords:** Resorbable polymers, Scaffolds, Soft tissue augmentation, Tissue engineering, 3D printing

## Abstract

**Objectives:**

Histological and histomorphometric evaluation of the efficacy of custom-made three-dimensional (3D) printed poly-lactic acid (PLA)/ polyethylene glycol (PEG) scaffolds in horizontal soft tissue augmentation compared to connective tissue grafts (CTG) in Mongrel dogs.

**Materials and methods:**

Horizontal ridge defects were created after tooth extraction in 12 dogs, then randomly allocated to one of the 3 study groups. 1 month after the first surgery, defects were augmented using custom-made 3D printed PLA/PEG scaffolds, or CTG or were left empty. 6 dogs were sacrificed after 1 month post-operatively, and 6 dogs after 3 months. Samples were collected for histologic evaluation.

**Results:**

PLA/PEG and CTG groups successfully augmented soft tissue thickness. Histomorphometrically, fibroblast count (FC) was significantly higher in PLA/PEG group at both time points in comparison to CTG (*p* = 0.002 and 0.007) and control groups (*p* = 0.001 and 0.016). Percentage of collagen surface area (%CSA) was significantly higher in PLA/PEG group compared to other groups at 1 month (*p* = 0.021 and 0.001), however, it was higher in CTG group at 3 months (*p* = 0.024 and 0.003).

**Conclusions:**

3D printed custom-made PLA/PEG scaffolds are an easy, effective, and safe method for soft tissue volume augmentation.

**Clinical relevance:**

The use of 3D printed scaffolds with properly designed internal structure guides the type of tissues regenerated and eliminates the need for CTG with its higher morbidity and complications.

**Supplementary Information:**

The online version contains supplementary material available at 10.1186/s12903-025-07051-6.

## Introduction

Tooth extraction causes resorption and remodeling of alveolar ridges, resulting in deficiencies that peak after 6 months especially in horizontal direction [1–3]. Tooth or implant-supported restorations require augmentation of these deficiencies for esthetic and/or functional purposes, yet complicate the treatment plan. Quality and quantity of available soft tissue can be improved through augmentation procedures, with autogenous grafts being the gold standard. However, complications like bleeding, infection, prolonged surgery and patient morbidity exist [4–6].

Tissue engineering (TE) is a science aiming to repair or restore lost tissues or organs. It comprises of 3 elements; cells, scaffolds and signaling molecules. Scaffolds are crucial for TE-based regenerative processes, maintaining its structural integrity and providing a stable surface for cells and growth factors attachment. They should mimic the three-dimensional (3D) architecture of native tissue’s extracellular matrix for structural, mechanical, and biological regeneration. Parameters like material crystallinity, strand diameter, pore size, interconnectivity, and total porosity control scaffold’s architecture. 3D printing offers higher accuracy and control of scaffold design over conventional fabrication methods [7–10].

Selection of scaffold material is crucial, with polylactic acid (PLA) being a popular choice due to its biodegradability, biocompatibility and mechanical properties. However, it has disadvantages like brittleness, slow degradation and hydrophobicity. Polyethylene glycol (PEG) is often used to overcome these issues, enhancing PLA’s properties through its hydrophilicity, biocompatibility, and biodegradability. These two polymers are mixed to create a hybrid filament, processed into composite scaffold with superior properties [11]– [12].

The main hypothesis was utilizing a scaffold with hybrid design where 3D printed PLA scaffold provides a stable framework and its injection with resorbable hydrophilic PEG gradually attracts cells through scaffold’s pores. The present study assessed histologically and histomorphometrically efficacy of 3D printed PLA/PEG scaffolds and compared it to CTG for soft tissue augmentation in dogs.

## Materials and methods

### Animal grouping and allocation

12 adult healthy Mongrel dogs (18–24 months, 10–18 Kg) were recruited in this study. 2 weeks before the experiment, animals were examined for health to ensure absence of systemic conditions. They were kept in separate kennels with 12:12 light/darkness cycle and proper ventilation and nutrition. Animals followed a soft food diet with unlimited water supply and were monitored daily. Animals were checked twice daily for appetite, wound healing, and overall condition by a veterinarian throughout the study period. All animals (males and females) were provided and housed by the experimental animal unit, tissue engineering laboratories, Faculty of Dentistry, Alexandria University. The study protocol received approval of the Research Ethics Committee of the Faculty of Dentistry, Alexandria University for the conduct of research on animals (IRB No. 001056- IORG 0008839).

The study was performed in two surgical phases following Animal Research: Reporting of In Vivo Experiments (ARRIVE) guidelines [13]: bilateral extraction of 2 premolars creating defects (phase I) followed by soft tissue augmentation 1 month later (phase II). A total of 48 defects (16 per group) were randomly allocated into 3 groups using a computer software (Excel; Microsoft, Redmond, USA) by an investigator not involved in the surgeries. They were augmented using either custom-made 3D printed PLA/PEG scaffolds (PLA/PEG group), CTG (CTG group) or were left empty (control group). All animals enrolled in the study were included in the analysis with no exclusions.

### Scaffold fabrication

#### Thermal analysis of PLA

Thermogravimetric analysis curves were recorded using Q500 TA Instruments (TA Instruments, New Castle, DA, USA), where nitrogen atmosphere heated samples from room temperature to 500 °C at a rate of 10 °C/min. PLA’s glass transition temperature (Tg), melting temperature (Tm), and degradation temperature were assessed [14].

#### 3D printing and customization of PLA scaffolds

**Fig. 1:**
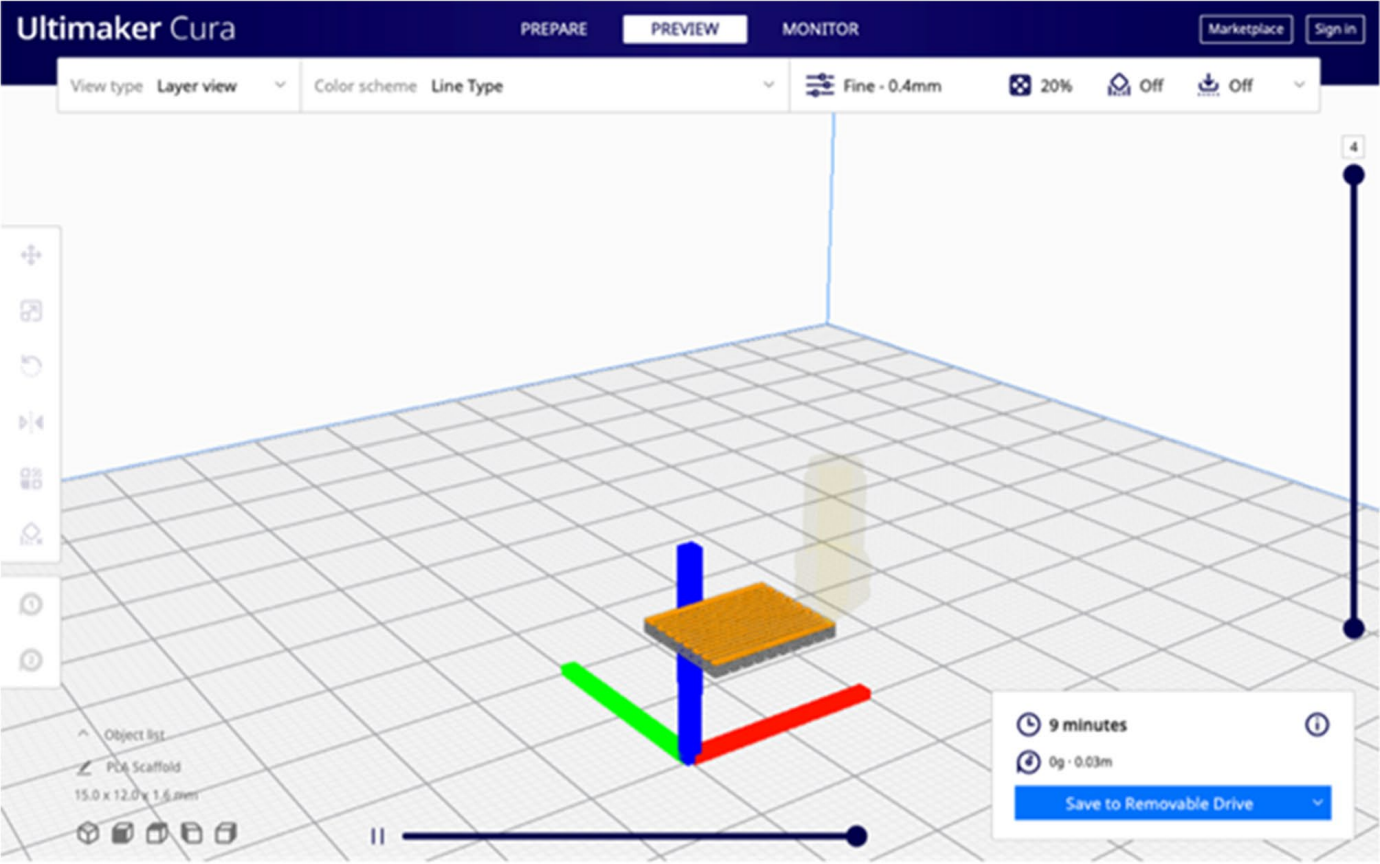
Design of 3D PLA scaffold on computer-aided design software

Computer-aided design software (Autodesk Tickercad, USA) was used to design a 3D model (15 mm x 12 mm x 1.6 mm). (Fig. 1) Open-source software (Cura Ultimaker version 4.11, USA) was used to set printing parameters (Table 1), slice STL file of 3D model and generate a G-code to be run by the printer for scaffold fabrication. Melt extrusion 3D printer (BIO 2, Robota, Egypt), was fed medical grade PLA pellets (Xiamen Keyuan Plastic Co.,Ltd, China) and extruded by mechanical piston pressure through syringe nozzle 0.5 mm in diameter.

Field emission scanning electron microscope (SEM) (JSM-5510LV, JEOL Ltd company, Tokyo, Japan) analyzed scaffolds. They were sputter coated with gold then images were obtained at an accelerated voltage of 10 KV. Pore size and morphology were measured using open-source software (Image J 1.53k, NIH, USA).

**Fig. 2:**
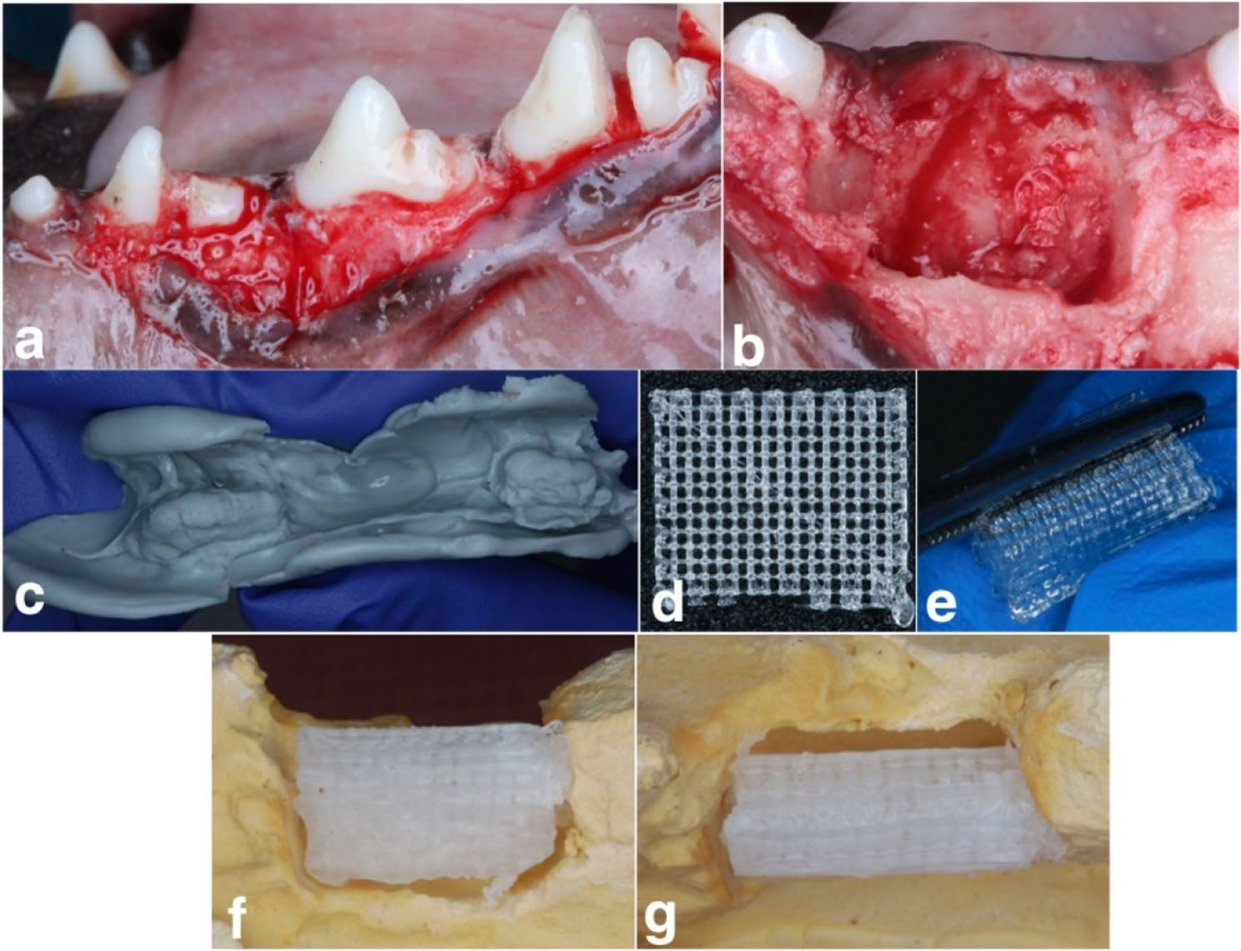
Creation of defects in phase I and scaffold customization. **A** Sectioning of mandibular P2 and P4 prior to extraction. **B** Extraction, defect creation and adjustment. **C** impression of defect. **D**3D printed PLA scaffold. **E**,**F**,**G** Customization and fitting of PLA scaffold on stone model + injection with PEG

Defects created (details in 2.4) in the first dog were used a reference. Shape of defects was recorded with addition silicone impression material (Zetaplus, Zhermak, Italy) and cast in extra hard dental stone (GCS Dent, Egypt) obtaining stone models. PLA scaffolds were customized on stone models by dipping them in a warm water bath set to Tg of PLA and folding it into a U-shape with 1 mm between the 2 layers. Additional adjustments were done using scissors until perfect fit was achieved. Scaffolds were sterilized by ultraviolet light [15]. PEG flakes (Oxford Laboratories Pvt. Ltd, Mumbai, India) were loaded in plastic syringe and heated in a water bath (50–60 °C) until melting then used to fill gaps between the 2 PLA layers (Fig. 2c-g).

**Table 1:** Melt extrusion printing parameters of PLA pellets

Printing process Parameters	PLA Polymer Pellets
Layer Height	0.4 mm
Line Distance	0.35 mm
Printing temperature	185 °C
Infill pattern	0º/90º
Printing speed	2 mm/sec

### Phase I (Extraction and defect creation)

For all interventions, after fasting for 12-hours, animals were sedated by intravenous propofol (Diprivan 1%, AstraZeneca UK, LTD, UK) (2 mg/kg) and general anesthesia was reached by 1.5–2.5% isoflurane (Isoflurane, ACDIMA Int., Egypt). Local infiltration anesthesia was done using 4% articaine hydrochloride (Artinibsa 40 mg/ml, Inibsa Dental, Spain) with 1:100000 adrenaline [16].

Surgery involved mucoperiosteal flaps reflection, mandibular second and fourth premolars (P2/P4) sectioning and extraction. Defects were enlarged into a rectangle and irrigated with saline. Defect measurements are provided as Supplementary Figure S1 Primary closure was achieved by interrupted and horizontal mattress sutures using polypropylene (2/0) sutures (Fig. 2a, b) [17].

### Phase II (Soft tissue augmentation) (1 month after phase I) (Fig. 3a-g)

PLA/PEG group involved fitting PLA/PEG scaffold in defects after mucoperiosteal flaps reflection and cortical perforations. Molten PEG was injected filling spaces between scaffold and bone. In CTG group, free gingival graft 3 mm in thickness was harvested from the lateral palatal vault, de-epithelialized to be transformed into CTG, folded in half and sutured, then immobilized in a partial thickness buccal pouch by a single cross-horizontal mattress suture [17]. For control group, no augmentation was received.

**Fig. 3:**
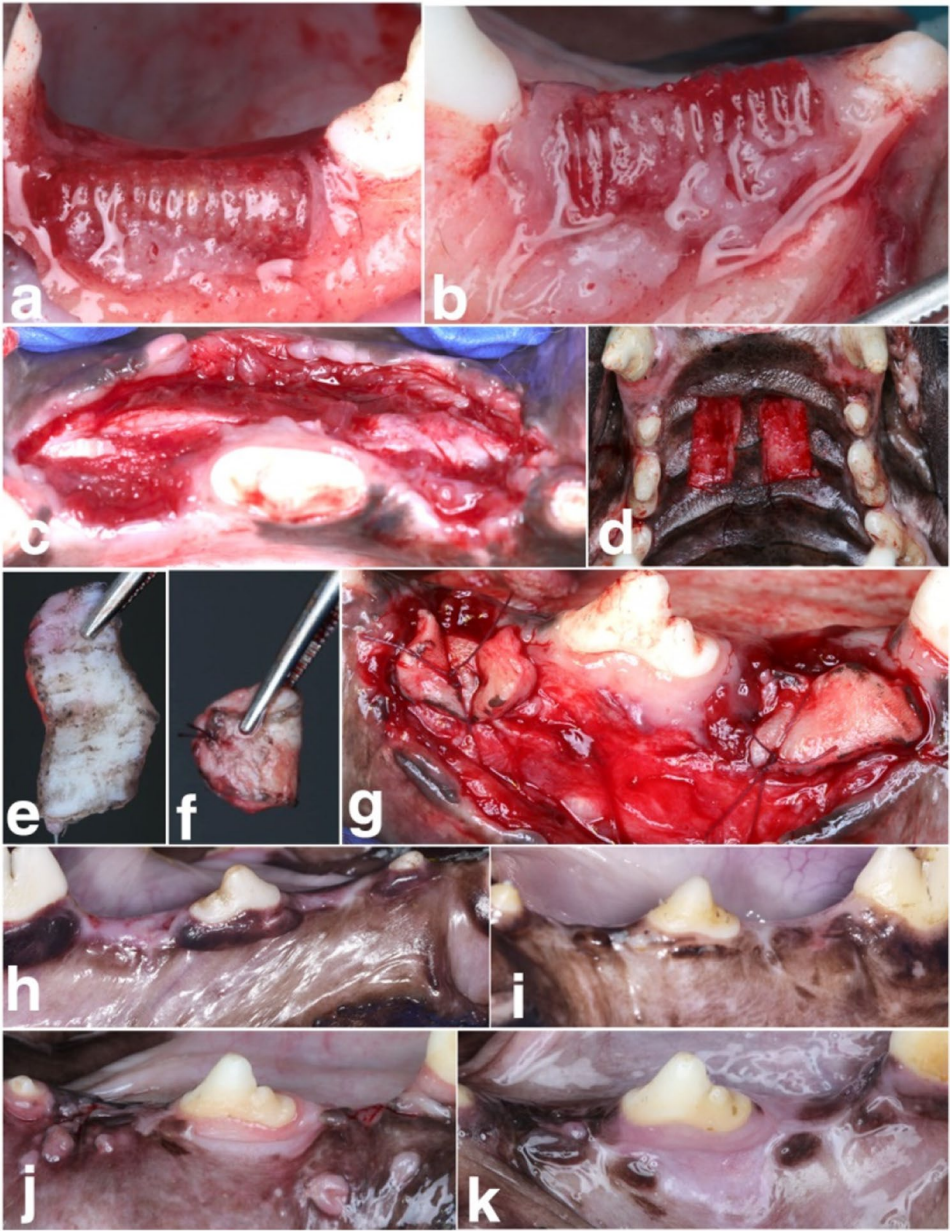
**A**,**B **Fitting of customized 3D printed PLA/PEG scaffold in defect. **C-G** partial thickness flap, harvesting of graft from the palate, de-epithelialization and folding of CTG and immobilization in defects. **H**,**I **Healing of PLA/PEG group at 1 and 3 months. **J**,** K **Healing of CTG group at 1 and 3 months

On the day of each surgery, animals received intramuscular 1 ml amoxicillin/clavulonic acid (Synulox 100 ml vial, Zoetis, Egypt) and diclofenac sodium (Voltaren 75 amp^®^, Novartis Co., Egypt) (1.1 mg/kg). Amoxicillin/clavulonic acid (Augmentin 500 mg, GlaxoSmithKline, Egypt) and non-steroidal anti-inflammatory drug (Meloxicam 3.5 mg, DELTA PHARMA Factory-Industrial Zone B4, Egypt) tablets were crushed and mixed with their food for 7 days [16].

Six dogs were euthanized 1 month after phase II and the remaining 6 after 3 months via an intravenous propofol overdose.

### Histologic and histomorphometric analysis

Specimens were fixed in 10% neutral buffered formalin, washed and decalcified using 5–10% trichloroacetic acid. Decalcified specimens were placed under running water, dehydrated in gradually increasing concentrations of ethanol, cleared in xylene, covered with soft paraffin wax and embedded in polymethacrylate blocks. Sections were stained with Haematoxylin and Eosin (H&E) stain after serial cuts of 4 μm thickness in the buccolingual direction were made with a rotary microtome.

Histologic examination and histomorphometric analysis were done by a single expert blinded to group allocation. Native and newly formed soft tissue, integration and vascularization of new tissues, and resorption of PLA scaffold were assessed at 1 and 3 months. Digital images were acquired by mounting a color CCD camera (Optikam B10, Optika S.r.l, Italy) on a binocular light microscope (Optika S.r.l, Italy). Images with (x40 and x100) magnifications were assessed using image analysis software.

Percentage of collagen fibers surface area (%CSA) (primary outcome) and residual scaffold material surface area (%RSSA) and fibroblast count (FC) (secondary outcomes) were calculated for all groups at 1 and 3 months. 2 sections from the center of the defect were obtained per specimen, and 4 fields were randomly selected per section, bringing the total to 8 measurements/section. A schematic diagram illustrating the region of interest is provided in Supplementary Figure S2. Mean value of measurements was recorded and used in statistical analysis. Image analysis software was calibrated and similarity between repeated measurements of the same section was at ˃95% level.

### Statistical analysis

G*Power software (G*Power, Ver. 3.192 copyright 1992–2014, Düsseldorf, Germany) was used for power analysis. Sample size was calculated based on previous studies [17, 18] and assuming 5% α-error and 80% power. The highest sample size was calculated based on differences between two independent means using standard deviation = 33.29, minimum sample size was 7 defects per group, increased to 8 defects to make up for processing errors. Total sample size was 48 defects in 12 dogs.

Data were analyzed using IBMSPSS^Ⓡ^ Statistics (IBM SPSS Statistics for Windows, version 20.0. Armonk, NY: IBM Corp, USA). Data normality was assessed using Shapiro–Wilk. Independent t-test was used for intragroup comparisons and One-way Analysis of Variance (ANOVA) test for intergroup comparisons for data with normal distribution (FC). Mann-Whitney test was used for intragroup comparisons and Kruskal-Wallis test followed by Dunn’s post hoc test for intergroup comparisons for data with non-normal distribution (%CSA and %RSSA). For all tests, values were expressed as means ± standard deviation and significance level was set at *p* ≤ 0.05. All the collected data were included in the analysis without any exclusions.

## Results

### Results of scaffold fabrication

#### Thermal analysis of PLA polymer

**Fig. 4:**
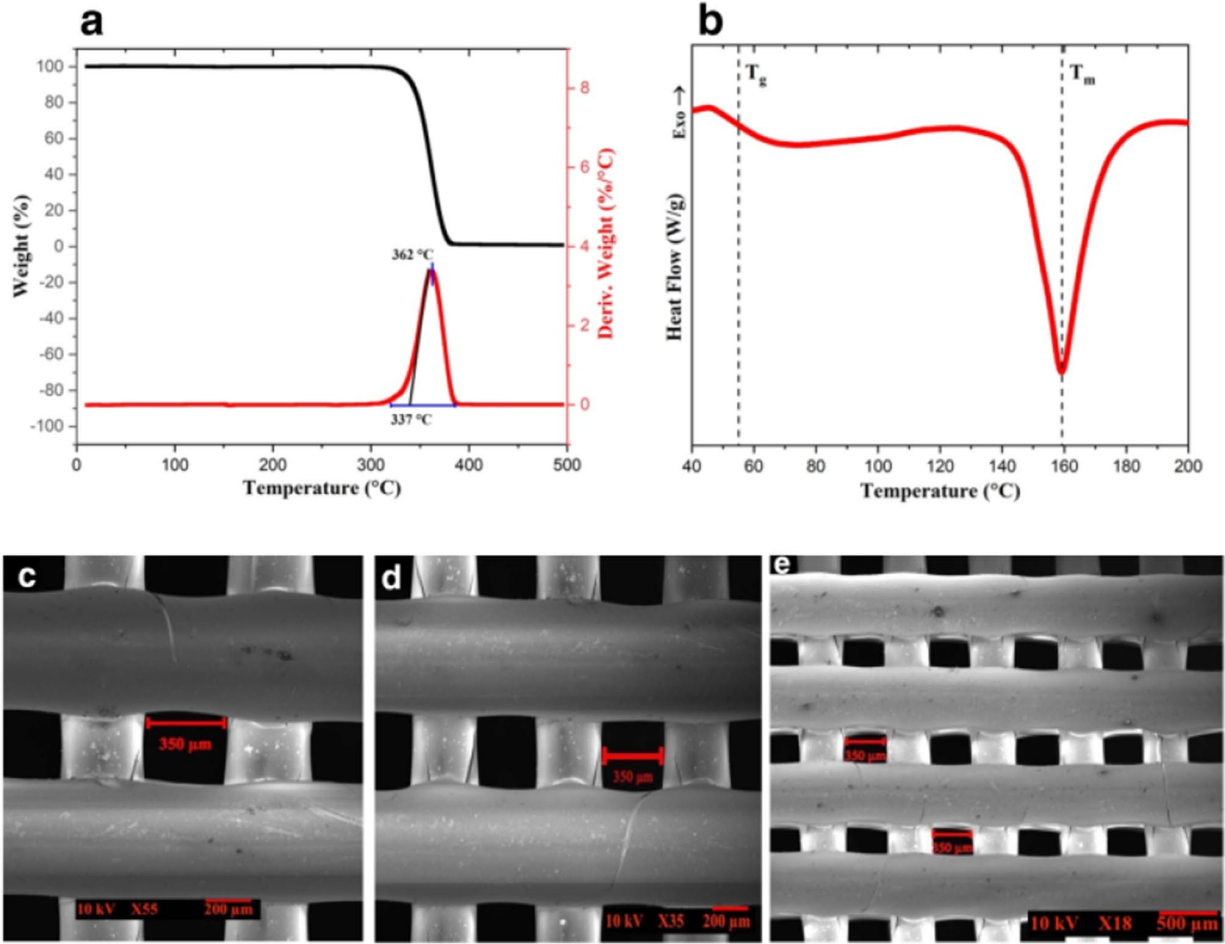
**A**Thermogravimetric, differential thermal gravimetry, and** B **differential scanning calorimetry curves of PLA polymer **C-E** SEM images of PLA scaffolds showing morphology and pore size at different magnifications (x55, x35 and x18 respectively)

Thermogravimetric, differential thermal gravimetry and differential scanning calorimetry curves for PLA are shown in Fig. 4a, b. Onset of PLA weight loss was at 337 °C while maximum degradation temperature was 362 °C. DSC results revealed T_g_ in the range of 55 °C, and T_m_ around 160 ° [19].

#### Morphology and microstructure

SEM micrographs at different magnifications are shown in Fig. 4c-e. A multi-layered connected structure of scaffolds was observed with a mean pore size of 350 ± 10 μm.

### Clinical findings

All animals tolerated all surgical procedures and survived the entire study period. No systemic or local complications were reported except one site demonstrating wound dehiscence after surgery 1 and was managed through degranulation, irrigation, and flap release before augmentation surgery. No exposure of scaffolds or CTG was observed. (Fig. 3h-k).

### Histologic evaluation

**Fig. 5:**
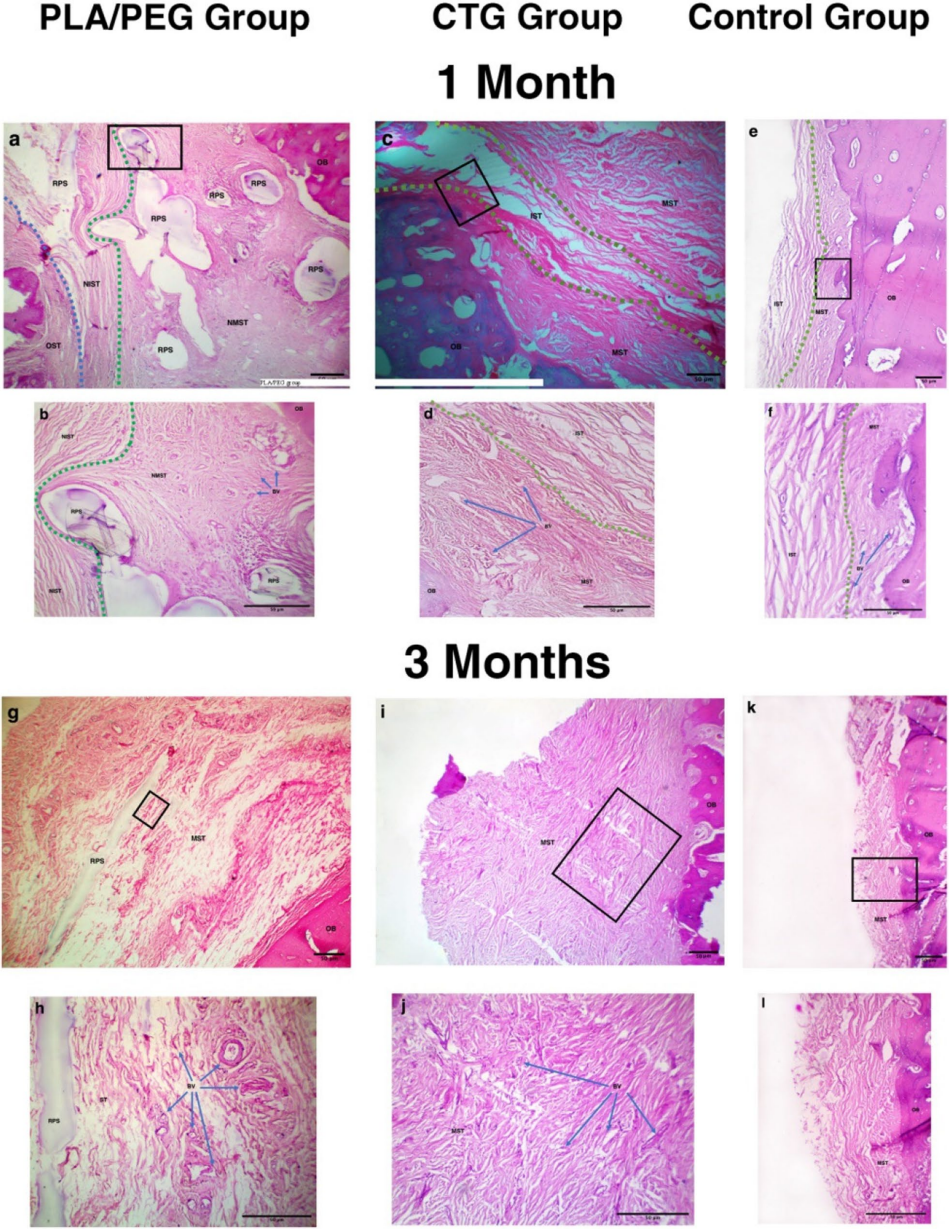
Histological evaluation of PLA/PEG, CTG and control groups, in soft tissue augmentation. **A**,**B** Photomicrograph of PLA/PEG group after 1 month showing interface between old bone (OB) and NMST (Blue dotted line). RPS with NIST above it and NMST underneath it. Line of demarcation between NIST and old soft tissue of buccal flap (OST) (Green dotted line). Blood vessels (BVs) highly concentrated in NMST (blue arrows). **C**,**D** Photomicrograph of CTG group after 1 month showing interface between OB and mature MST. Area of loosely arranged IST between 2 areas of dense MST (between the 2 dotted green lines). Complete integration between CTG and native soft tissue. BVs highly concentrated in MST. **E**,**F **Photomicrograph of control group after 1 month showing interface between OB and MST. IST overlying MST and significantly reduced thickness in comparison to other 2 groups. **G**,**H** Photomicrograph of PLA/PEG group after 3 months showing less RPS and MST completely integrated with OB and OST. Larger number of BVs with equal distribution. **I**,**J** Photomicrograph of CTG group after 3 months showing well-arranged MST. Equal distribution of BVs. **K**,**L **Photomicrograph of control group after 3 months showing OB covered by MST and overall reduced soft tissue thickness. (All sections are stained with H&E × 40 and insets (black rectangles) are H&E× 100) (*OB* old bone, *NMST* new mature soft tissue, *RPS* residual PLA scaffold, *NIST* new immature soft tissue, *OST* old soft tissue, *BV* blood vessels, *MST* mature soft tissue, *IST* immature soft tissue)

Both augmentation modalities in PLA/PEG and CTG groups successfully formed new soft tissue and increased gingival thickness (Fig. 5).

At 1 month in PLA/PEG group, the scaffold began to degrade into capsules with remnants of PLA scaffold (RPS) randomly distributed. A dense network of organized newly formed soft tissue replaced the degrading scaffold, with new mature soft tissue (NMST) towards the bone and new immature soft tissue (NIST) towards the buccal flap. NIST bundles had a parallel pattern of organization and was fully integrated with NMST. A line of demarcation can be seen between NIST and the overlying native buccal flap tissues. Several blood vessels densely arranged around RSM and NMST could be observed throughout the sections. (Fig. 5a, b) At 3 months, PLA scaffolds showed further degradation and replacement by mature soft tissue (MST). MST had densely packed well-arranged collagen bundles fully integrated with buccal flap. Blood vessels were smaller in diameter and less engorged with blood and equally distributed throughout the slides. (Fig. 5 g, h).

At 1 month in CTG group, MST with dense collagen fibers was arranged near surface epithelium and bone with immature soft tissue (IST) in between. It was very difficult to differentiate between native and augmented tissues. Blood vessels evenly distributed but more concentrated around MST. (Fig. 5c, d) At 3 months, MST had denser, more mature and better organized bundles. A higher number of blood vessels was observed. (Fig. 5i, j) Control group exhibited findings similar to group II but with less tissue thickness. (Fig. 5e,f,k,l)

### Histomorphometric analysis

Regarding FC, there was a statistically significant higher number in all groups at 1 month in comparison to 3 months (*p* = 0.0082, 0.001 and 0.026 respectively). PLA/PEG group exhibited a statistically significant higher count at 1 and 3 months compared to CTG group (*p* = 0.002, 0.007 respectively) and group III (*p* = 0.001, 0.016 respectively). CTG group had a statistically significant higher count at 1 month compared to control group and an insignificant difference at 3 months (*p* = 0.016 and 0.103 respectively).For %CSA, there was a statistically significant difference in all study groups in favor of 3 months compared to 1 months (*p* = 0.001). At 1 month, there was a statistically significant difference between PLA/PEG group compared to CTG and control groups (*p* = 0.021, 0.001 and 0.001 respectively). However, at 3 months the significant difference was in favor of CTG group compared to PLA/PEG and control groups (*p* = 0.024, 0.003 respectively). Also, PLA/PEG group has a significant difference compared to control group (*p* = 0.001).

**Fig. 6:**
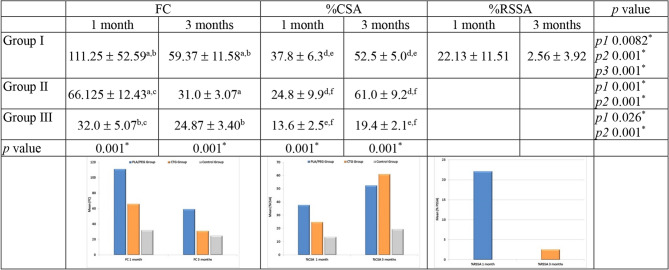
Histomorphometric outcomes. ^*^Significant difference between the 3 groups (*p≤*0.05). *p1* comparison between 1 and 3 months for FC *p2 *comparison between 1 and 3 months for %CSA *p3* comparison between 1 and 3 months for %RSSA. ^a^Significant difference (0.002, 0.007) between group I and II at 1 and 3 months for FC. ^b^Significant difference (0.001, 0.016) between group I and III at 1 and 3 months for FC. ^c^Significant difference (0.016) between group II and III at 1 month for FC. ^d^Significant difference (0.021, 0.024) between group I and II at 1 and 3 months for %CSA. ^e^Significant difference (0.001, 0.001) between group I and III at 1 and 3 months for %CSA. ^f^Significant difference (0.013, 0.003) between group II and III at 1 and 3 months for %CSA

In PLA/PEG group, there was a statistically significant reduction in %RSSA between 1 and 3 months (*p* = 0.0001) (Fig. 6).

## Discussion

The present study aimed to evaluate and compare histologic and histomorphometric effects of custom-made 3D printed PLA/PEG scaffolds and CTG for soft tissue augmentation of chronic defects in a dog model. It revealed that (i) 3D printed PLA/PEG scaffolds successfully increased soft tissue thickness, (ii) results were comparable to CTG regarding quality, quantity, integration and vascularity of new tissues at all follow-ups and (iii) scaffolds gradually degraded to be replaced by new soft tissue throughout the study period.

Scaffolds are abundantly used in TE to regenerate lost tissues in several medical fields [7, 8]. To the best of our knowledge, no other group has attempted to use custom-made 3D printed PLA/PEG scaffolds to augment soft tissues. However, similar hypothesis was implemented for the regeneration of other types of soft tissues.

A canine model was used due to scarcity of data about this methodology and their faster regenerative capacity. Average defect dimensions in the literature were used as reference for standardized scaffold dimensions (12 mm x15 mm x 1.6 mm) [16, 20]. Scaffold customization was carried out within the Tg of PLA, confirmed through thermogravimetric analysis. It allowed easy deformation while in the rubbery state before returning to the glassy state without fracture, resulting in U-shaped exoskeleton of 4.5 mm thickness.

PLA was the material of choice because of its FDA approval, biocompatibility, biodegradability and excellent mechanical properties. Although degradation time is long (< one year), it did not present much of a problem. The sole disadvantage was hydrophobicity; however, this can be readily mitigated by amalgamating it with more hydrophilic polymers, such as PEG, utilized in our work [12].

The scaffolds’ interconnected pores sized 350 ± 10 μm, were initially blocked with PEG. When in contact with blood from cortical perforations, PEG gradually dissolves, allowing cells (smaller then larger) and blood to pass through the pores and reach the center. Osteoblasts were excluded due to their size, and scaffolds are already saturated by other smaller cells. This solution addressed PLA hydrophobicity and issues of nutrient and oxygen supply.

3D printing was used for scaffold fabrication because it can produce exact replicas of specified patterns, facilitating accurate design and regulating macro- and micro-architecture, augmenting their regenerative capacity [10].

At 1 month, PLA/PEG group showed NIST easily demarcated from surrounding mature tissues, indicating a pattern of maturation from deeper to superficial layers. The tissues closer to the bone were influenced by blood clot from cortical perforations. The tissues closer to buccal flap provided another source of cells, supplying the scaffold with cells from all directions. Scaffolds showed a degradation pattern, deducted from distribution of RPS and new tissue formation, starting near lingual bone and progressing towards buccal flap tissues, gradually replaced by new soft tissue. At 3 months, new MST was integrated into buccal flap tissues. Collagen fibers were arranged in parallel layers which is thought to be related to their conforming with scaffold internal architecture. Also, a significant reduction in %RSSA was noticed confirming its biodegradability. Another interesting observation is good vascularization of all sections at both time points, but at 1 month, blood vessels were more around RPS with an equal distribution within newly formed tissues at 3 months. This is thought to be the result of the scaffold’s interconnected pores promoting initial angiogenesis and invasion of cells which gradually decreased due to lesser need for nutrition.

These histologic findings highly were in accordance with a study by Meng et al. [21] who implanted 3D printed hybrid scaffolds subcutaneously in rats to regenerate adipose tissue. They succeeded in formation of well-aligned fibrous tissue, new blood vessels, adipose tissue infiltration and gradual degradation of the scaffold. In addition to Dong et al. [22] who implanted 3D printed poly-4-hydroxybutyrate in vivo for nipple reconstruction and successfully achieved infiltration and maturation of adipose-rich fibrous tissue.

Histomorphometrically, PLA/PEG group showed decrease in FC and increase in %CSA at 3 months, attributed to initial high fibroblasts influx from blood clot and buccal flap connective tissue and infiltration scaffold pores. As healing progressed, collagen fibers matured, increasing %CSA and decreasing FC. CTG group had less FC and %CSA at 1 month, but less FC and more %CSA at 3 months compared to PLA/PEG group. This can be attributed to RPS that may be replaced by new tissues and different healing mechanisms between 2 groups, where PLA/PEG group had a process previously explained so %CSA may still increase with longer healing period, with less possibility of change, while CTG group required only remodeling of the graft [20].

Very close similarities were found by Thoma et al. [23] and Naenni et al. [20] where soft tissue was augmented using collagen matrices. Collagen matrices, especially cross-linked types acted like scaffolds and stimulated fibroblast influx, vascularization and deposition of collagen fibers. However, the comparison is not fair due to different materials used. Moreover, Ovcharenko et al. [24] found that bioresorbable porous PLA membrane used to cover extraction sockets was accepted by native tissues without adverse effects and resulted in significant soft tissue thickness gain. Petposri et al. [25] used 3D printed polylactic acid-glycolic membranes, very close in structure to our scaffold, for guided bone regeneration and concluded that membranes with low GA ratios had higher attachment density of fibroblasts and osteoblasts, while the high GA ratio group had lower attached cells. They also reported degradation period of 8 weeks which aligns with our study.

The study showed promising results but had limitations: (i) scaffold manipulation process was delicate due to PLA’s brittle nature, rendering it prone to breakage, (ii) the impregnation with molten PEG required speed and careful handling, (iii) minor scaffold adjustments were sometimes required to accommodate defects’ dimensional changes between surgeries 1 and 2 and (iv) volumetric analysis was not performed and our conclusions were only based on histomorphometric indicators (%CSA and FC). It can be concluded that our scaffold is a safe, easy, and effective alternative to CTG for increasing soft tissue volume. Future studies incorporating volumetric imaging and human clinical trials are warranted to validate and expand upon these findings.

## Supplementary information

Supplementary material 1.

Supplementary material 2.

Supplementary material 3.

## Data Availability

All data included in this current study are available from the corresponding author upon request.

## Abbreviations

TE: Tissue engineering
PLA: Polylactic acid
PEG: Polyethylene glycol
CTG: Connective tissue graft
TG: Glass transition temperature
TM: Melting temperature
SEM: Scanning electron microscope
H&E: Haematoxylin and eosin
%CSA: Percentage of collagen fibers surface area
%RSSA: Residual scaffold material surface area
FC: Fibroblast count
RPS: Remnants of PLA scaffold
NMST: New mature soft tissue
NIST: New immature soft tissue
MST: Mature soft tissue
IST: Immature soft tissue
