# Supplementary figures and images for: Evaluation of custom-made 3D printed polylactic acid/polyethylene glycol scaffolds in soft tissue augmentation: an experimental study in a canine model
Source: BMC Oral Health. 2025 Oct 27;25:1685. doi: 10.1186/s12903-025-07051-6 (PMC12560599; doi:10.1186/s12903-025-07051-6)

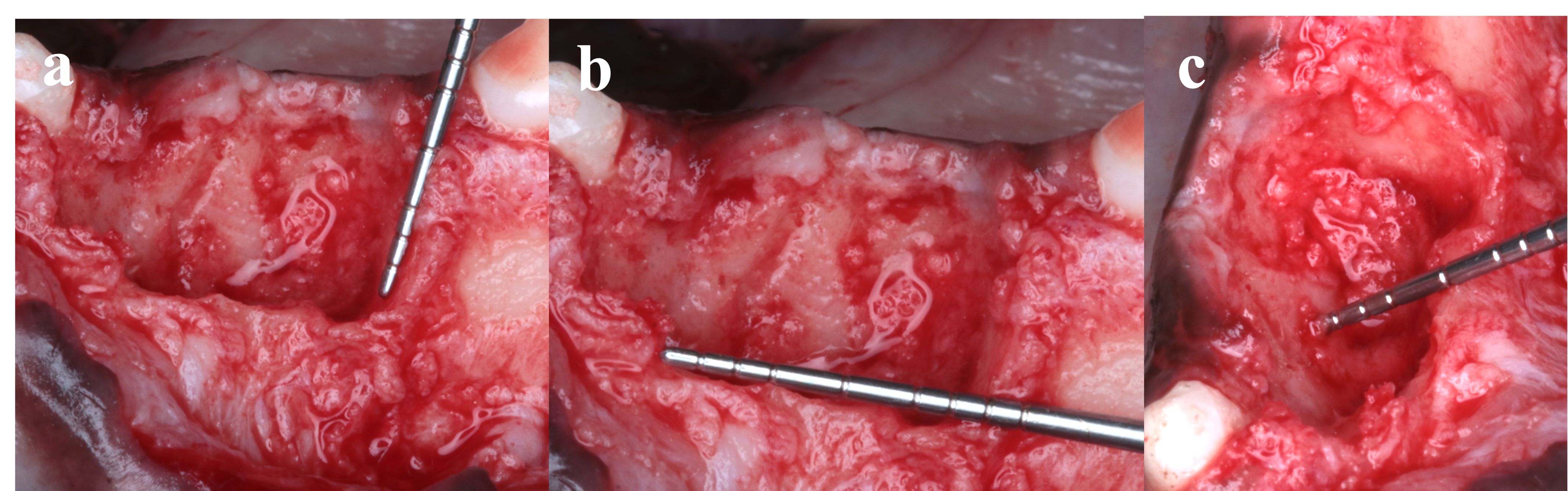

Supplement: Supplementary file 1 — Supplementary material 1. [file 12903_2025_7051_MOESM1_ESM.png]

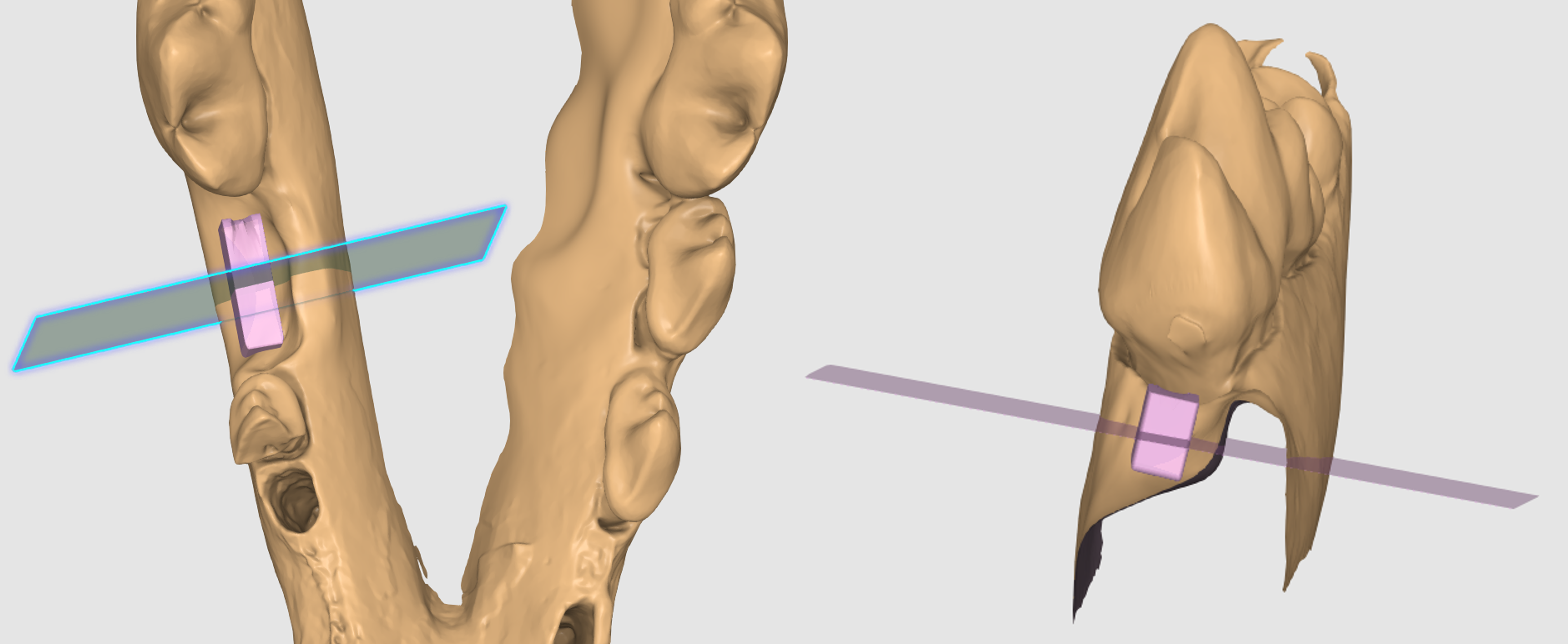

Supplement: Supplementary file 2 — Supplementary material 2. [file 12903_2025_7051_MOESM2_ESM.png]
