# Supplementary material for: Evaluation of custom-made 3D printed polylactic acid/polyethylene glycol scaffolds in soft tissue augmentation: an experimental study in a canine model
Source: BMC Oral Health. 2025 Oct 27;25:1685. doi: 10.1186/s12903-025-07051-6 (PMC12560599; doi:10.1186/s12903-025-07051-6)
Supplement: Supplementary file 3 — Supplementary material 3. [file 12903_2025_7051_MOESM3_ESM.docx]

**Supplementary Figure S1:** Defect measurments

**Supplementary Figure S2:** Schematic diagram illustrationg hustological sectioning
